# An Integrated In Vivo/In Vitro Protein Production Platform for Site-Specific Antibody Drug Conjugates

**DOI:** 10.3390/bioengineering10030304

**Published:** 2023-02-28

**Authors:** Jeffrey Hanson, Dan Groff, Abi Carlos, Hans Usman, Kevin Fong, Abigail Yu, Stephanie Armstrong, Allison Dwyer, Mary Rose Masikat, Dawei Yuan, Cuong Tran, Tyler Heibeck, James Zawada, Rishard Chen, Trevor Hallam, Gang Yin

**Affiliations:** Sutro Biopharma Inc., 111 Oyster Point, South San Francisco, CA 94080, USA

**Keywords:** *E. coli* expression, cell-free protein synthesis, light chain, site-specific ACDs, antibody drug conjugate, cell-free manufacturing, titer improvement

## Abstract

The XpressCF+^®^ cell-free protein synthesis system is a robust platform for the production of non-natural amino acids containing antibodies, which enable the site-specific conjugation of homogeneous antibody drug conjugates (ADCs) via click chemistry. Here, we present a robust and scalable means of achieving a 50–100% increase in IgG titers by combining the high productivity of cell-based protein synthesis with the unique ability of XpressCF+^®^ reactions to produce correctly folded and assembled IgGs containing multiple non-natural amino acids at defined positions. This hybrid technology involves the pre-expression of an IgG light-chain (LC) protein in a conventional recombinant *E. coli* expression system, engineered to have an oxidizing cytoplasm. The prefabricated LC subunit is then added as a reagent to the cell-free protein synthesis reaction. Prefabricated LC increases IgG titers primarily by reducing the protein synthesis burden per IgG since the cell free translation machinery is only responsible for synthesizing the HC protein. Titer increases were demonstrated in four IgG products in scales ranging from 100-µL microplate reactions to 0.25-L stirred tank bioreactors. Similar titer increases with prefabricated LC were also demonstrated for a bispecific antibody in the scFvFc-FabFc format, demonstrating the generality of this approach. Prefabricated LC also increases robustness in cell-free reactions since it eliminates the need to fine-tune the HC-to-LC plasmid ratio, a critical parameter influencing IgG assembly and quality when the two IgG subunits are co-expressed in a single reaction. ADCs produced using prefabricated LC were shown to be identical to IgGs produced in cell-free alone by comparing product quality, in vitro cell killing, and FcRn receptor binding assays. This approach represents a significant step towards improving IgG titers and the robustness of cell-free protein synthesis reactions by integrating in vivo and in vitro protein production platforms.

## 1. Introduction

Antibody drug conjugates (ADCs) offer a means to specifically target cytotoxic compounds to tumors and are a promising modality for cancer therapy. Recent technological improvements addressing the limitations of the first generation of ADCs are due to a renewed interest in this class of molecules [1]. These improvements include new classes of potent cytotoxic drugs, improved linker stability, and the advancement of antibodies and conjugation methods [1,2]. These innovations in ADC technology provide a widened therapeutic index through improved potency, stability, and safety. Site-specific conjugation approaches are of particular interest since they enable homogeneous conjugates, which leads to molecules with greatly improved biophysical and pharmacokinetic profiles compared to heterogeneous conjugates produced by conventional random conjugation methods [3,4].

One of the most promising technologies for producing homogenous, site-specific ADCs is the incorporation of non-natural amino acids (nnAAs) into the IgG [5,6]. These novel amino acids can then provide an orthogonal chemical functional group, such as an aldehyde or azide, to which a drug-linker can be attached via click chemistry [5]. Unlike enzymatic conjugation methods, which have sequence motif or conjugation site requirements, nnAAs offer improved flexibility since they are encoded at the genetic level and can be inserted at almost any location in the protein with minimal perturbation to the surrounding sequence [3,7]. This method allows for fine tuning of the ADC conjugation sites, enabling the selection of the molecule with the desired efficacy, stability, and safety profiles.

Cell-free protein synthesis (CFPS) systems are uniquely suited for the production of nnAAs containing IgGs since they can be engineered to support high-fidelity amber suppression at multiple sites in a single poly-peptide chain, while their open nature allows for the establishment of an oxidizing environment suitable for IgG production and assembly [8]. Synthesis of nnAAs containing IgGs in mammalian systems is well established; however, amber suppression efficiency is limited by competition with the release factor (RF) responsible for translation termination at the TAG amber stop codon [9,10]. Efficient amber suppression of multiple nnAAs is achieved in *E. coli* by knocking out the RF which competes with amber suppression [11,12]; however, the reducing cytoplasmic environment is unsuitable for the production of properly folded and assembled IgGs at industrially relevant scales [13]. In addition, RF1 attenuation in vivo is complicated since it is required for cell viability in most *E. coli* strains. In contrast, cell-free expression systems offer a simple but robust solution to this challenge by taking advantage of the fact that CFPS is fully decoupled from cell growth. In one study, an outer membrane protease (OmpT) cleavage site was engineered into the switch loop of RF1 to enable its conditional inactivation after cell lysis [6]. The function of RF1 is preserved during cell growth, and then deactivated by OmpT upon lysis of the cell wall. The RF1 depleted extract allows nnAA incorporation at the previously intractable sites of an IgG, as well as at multiple sites in the same polypeptide chain. *E. coli*-based, cell-free protein synthesis is the preferred platform for the production of ADCs with a high drug-to-antibody ratio (DAR) since it combines the ability to incorporate multiple nnAAs with high efficiency after RF1 engineering, while the open nature of the cell-free system allows for a proper oxidizing environment for IgG folding and assembly [14,15].

**Figure 1:**
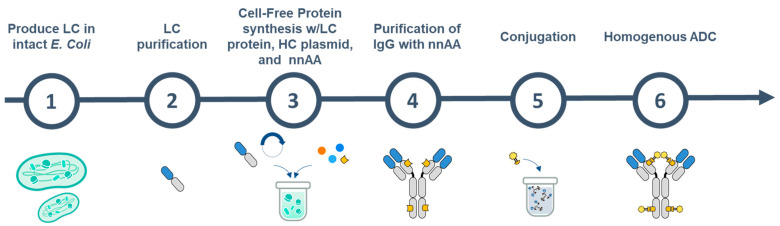
Schematic showing production of homogenous ADCs using the integrated in vivo/in vitro protein production platform, where LC protein is first produced in intact *E. coli* cells, purified, and added as a reagent to a cell-free protein synthesis reaction containing HC plasmid and non-natural amino acids (nnAAs). Purified IgG is subsequently conjugated to a cytotoxic liker-warhead using site-specific click chemistry in order to produce a homogenous ADC.

One of the challenges facing cell-free protein synthesis as a manufacturing platform is its lower productivity relative to conventional protein expression systems such as *E. coli* and CHO. Efforts to maximize CFPS productivity typically focus on changes in the strain [16,17], extract process [18], or composition of the cell-free reaction [19,20,21]. In the present work, we demonstrate a novel method for improving cell-free volumetric titers: adding a subunit of the target protein as a reagent, thereby reducing the protein synthesis burden for each molecule produced in the CFPS system. To date, cell-free IgG production has been achieved by co-expression of the HC and LC from two plasmids added individually to the CFPS reaction [14]; however, the two IgG subunits behave differently when expressed individually in the CFPS reaction. LC proteins are expressed solubly and are correctly folded, whereas the HC tends to form insoluble aggregates in the absence of the LC. We have previously shown that the addition of the LC plasmid prior to the HC plasmid—effectively pre-expressing the LC protein in cell-free reactions—improves IgG folding and assembly [14]. In addition, we have shown that prefabricated LC can be purified from CFPS reactions and added back to cell-free reactions expressing the HC fragments for high-throughput ribosome display [22]. Here, we developed an *E. coli* host strain with an oxidizing cytoplasmic environment capable of expressing soluble LC at high levels. Purified LC protein was then added to CFPS reactions expressing the HC protein only (Figure 1). This hybrid in vivo/in vitro protein production platform leverages the advantages of both recombinant *E. coli* expression (high titers and simple manufacturing) and cell-free protein synthesis (incorporation of multiple nnAAs in a correctly assembled IgG) to achieve a 50–100% increase in IgG titers using the CFPS platform. This technology not only increases titers in CFPS reactions, resulting in a lower cost of goods, but also improves the robustness of the cell-free reaction, streamlining and simplifying the manufacturing process.

## 2. Materials and Methods

### 2.1. Light-Chain Construct Design

To produce plasmid for the expression of LC in the *E. coli* cytoplasm, the genes for the mature LC, codon-optimized for expression in prokaryotic systems, were synthesized using ATUM and cloned into the vector pJ411. The resulting pJ411-LC vectors have a T7 promoter and T7 terminator for high-level LC transcription. This plasmid has a high copy pUC origin of replication and contains a Kanamycin selectable marker.

### 2.2. Strain Engineering

Genomic manipulations, including knockins and knockouts, were performed with a modified site-specific recombination protocol [23]. These methods utilize homologous recombination to specify the exchange sites within the chromosome. Site-specific recombination involves specific, inverted repeat sequences, e.g., the Cre-LoxP systems. For insertions, the integration cassette was composed of the gene, e.g., *dsbC*, to be inserted adjacent to a selectable marker that was flanked by loxP sites. Mutants with the desired chromosomal modification were selected using antibiotic resistance. The selectable marker gene was subsequently eliminated from the chromosome by transient exposure to transformed plasmid with a heat-sensitive origin of replication encoding Cre recombinase.

### 2.3. Light-Chain Shake Flask Expression

The *E. coli* strains for LC expression were made by transforming an *E. coli* host strain with plasmid pJ411-LC. Strains were grown overnight at 37 °C in terrific broth (TB) with 50 µg/mL kanamycin (Kan). In the morning, the cultures were diluted 1:50 into fresh TB+Kan and grown at 37 °C to an OD_600_ of 1.5. At this point, the expression of the LC gene was induced by the addition of arabinose to a final concentration of 0.2%. The temperature was then adjusted to 25 °C and protein expression continued for 16 h. Cells were harvested by centrifugation at 6000× *g* for 10 min.

### 2.4. Light-Chain Bioreactor Expression

The fermentation process began by taking a 1-mL cell bank vial and inoculating a 250-mL baffled shake flask with 35 mL of the proprietary I17-SF defined media, containing glucose, amino acids, salts, vitamins, and trace metals, plus 30 µg/mL kanamycin. The culture was grown in a shaking incubator at 37 °C and 250 RPM until OD_595_ reached 2–4. The seed culture was used to inoculate a Sartorius Biostat Qplus 500-mL bioreactor at a seeding density of 1.5% (v/v) with 250 mL of batched media, which consisted of 0.1% (v/v) polypropylene glycol 2000 (P2000) antifoam and 1.2% (v/v) proprietary 10 × I17 defined media, which have the same components as I17-SF at concentrated levels and purified water. The initial bioreactor temperature, dissolved oxygen (DO), pH and agitation speed setpoints were 37 °C, 30%, 7.0, and 200 RPM, respectively. The initial total gas flow was set to 0.2 SLPM of air and then increased to 0.4 SLPM of air once DO reached the 30% setpoint. The DO setpoint was maintained by cascading the agitation first until it reached a max of 1200 RPM, and then increasing the percentage of pure oxygen in the total gas flow as needed. The pH was controlled using 10% (v/v) ammonia and 5% (v/v) phosphoric acid.

The production fermentation consisted of the batch, fed-batch, and induction phases. Once the cells grew to an OD_595_ between 2 and 5 in the batch phase, the fed-batch phase began by feeding 10 × I17 defined media at an exponential rate of 0.30 h^−1^ with an initial rate of 15 µL/min. After 10 h in the fed-batch phase, the temperature of the bioreactor was decreased to 25 °C and the exponential feed rate was decreased to 0.02 h^−1^. An hour later, the induction phase began by adding 2.5 mL of a 400 g/L L-Arabinose stock solution. The induction phase lasted 24 h.

At the end of the fermentation, the culture was collected and centrifuged at 18,592× *g* and 2–8 °C for 15 min in a floor centrifuge. The supernatant was discarded, and the cell pellets were resuspended and washed with S30 buffer solution (10 mM Tris acetate, pH 8.2, 14 mM magnesium acetate, and 60 mM potassium acetate) at a concentration of 16.67% (w/w) and centrifuged again under the same conditions used in the initial harvest step. After the wash centrifugation, the supernatant was discarded and the cells were resuspended with S30-5 buffer solution (10 mM Tris acetate, pH 8.2, 2 mM magnesium acetate, and 500 mM potassium acetate) at a concentration of 16.67% (w/w). The cell resuspension was then passed through an Avestin Homogenizer (EmulsiFlex-C5) at 17,000 psi to disrupt the cells and generate the crude lysate. The crude lysate was immediately chilled in an ice bath and further clarified by centrifuging at 18,000–20,000× *g* and 2–8 °C for 30 min in a floor centrifuge. 

### 2.5. Light-Chain Purification

Cell lysate was flocculated with the addition of polyethyleneimine (PEI, linear, M.W. 25,000) to a final concentration of 0.01% and clarified by centrifugation at 10,000 rpm for 30 min. The supernatant then underwent sterile filtration through a 0.8/0.2 micron filter with a polyethersulfone membrane and was captured on a Protein L (Cytiva) column equilibrated with 8 mM sodium phosphate and 137 mM sodium chloride, at pH 7.3. After the completion of the sample load, a 15-CV wash step with an equilibration buffer was applied to wash away unbound and weakly bound impurities. The bound PFLC was eluted with 50 mM acetic acid, pH 3.2 and neutralized to pH 8.0 with 1 M Tris base, at pH 9.5.

### 2.6. mAb Expression in Cell-Free Reactions

Cell-free reactions were prepared through the addition of 37.5% S30 extract [24], 50% 2x supermix [20], 3 µg/mL total plasmid, and 2 mM para-azidomethyl-l-phenylalanine (pAMF). *M. jannaschii* pAMF tRNA synthetase [5], *M. jannaschii* amber suppressor tRNA [5], and T7 RNA polymerase were individually over-expressed in intact *E. coli* cells and added to the CF reaction as a crude lysate at a final concentration of 1–2% each. For C^14^ reactions, 2% v/v C^14^-Leucine (Perkin Elmer, NEC279E001MC) was added to each reaction. Reactions of 100 µL were carried out for 16 h at 25 °C in a V-bottom 96-well plate with rotation at 650 rpm in a thermomixer R (Eppendorf). Analysis of the total and soluble IgG titer by C^14^ was performed as has been described previously [20]. Reactions of 1 mL were carried out in flowerplates (M2P Labs, MTP-48-OFF) at 25 °C with rotation at 650 rpm in a thermomixer R (Eppendorf).

Larger reactions were carried out at a 0.25-L scale in a bioreactor (DASgip, Eppendorf, Hamburg, Germany) with temperature, pH, and DO control. pH was maintained at 7.5 for 14 h using 1 M citrate and 1 M KOH. DO was maintained at 20% for 14 h by blending N_2_ and O_2_ gas. After 14 h, the pH and DO were changed to 8.0 and 80%, respectively, for three hours. IgG titer was determined by A_280_ after purification with PhyNexus Phytip ProPlus columns.

### 2.7. mAb Purification and Conjugation

CFPS reactions were harvested by centrifugation and the supernatant was captured with Protein A affinity resin (MabSelect Sure, Cytiva, Marlborough, MA, USA) and eluted with 100 mM Glycine at pH 3.2. The neutralized ProA pool was concentrated and polished by preparative SEC (Superdex 200) in phosphate-buffered saline (PBS). Product quality was accessed by analytical SEC (Zenix-C SEC-300, 3 μM, 7.8 × 300 mm, Sepax Technologies, Newark, Delaware, DE, USA) with a mobile phase of 50 mM sodium phosphate, 140 mM NaCl, and 5% isopropanol, at pH 6.5.

Polished IgG was diluted to 1 mg/mL in PBS and incubated at room temperature overnight with three-fold the molar excess (40 μM) of the DBCO-maytansine drug-linker SC236. Excess un-reacted drug-linker was removed by desalting spin column (Zeba spin desalting plate, 7K MWCO, Thermo Fisher Scientific, Waltham, MA, USA) equilibrated with PBS. Product quality was accessed by analytical SEC, and the Drug Antibody Ratio (DAR) was determined by MALDI-TOF MS with a Super-DHB matrix.

### 2.8. In Vitro Cell Killing Assays

The cytotoxicity effects of the ADCs were measured with a cell proliferation assay using SU-DHL-6 (ATCC), K562 (ATCC), and OPM-2 (DSMZ) cell lines. A total of 12,500 cells at a volume of 25 μL were seeded in a 384-well flat-bottom white polystyrene plate on the day of the assay. Filtered sterilized samples were serially diluted (1:3) under sterile conditions and added onto cells in quadruplicates. Plates were cultured at 37 °C in a CO_2_ incubator for 72 h. For cell viability measurements, 30 μL of Cell Titer-Glo^®^ reagent (Promega Corp, Madison, WI, USA) was added into each well, and the plates were processed as per product instructions. Relative luminescence was measured on an ENVISION^®^ plate reader (Perkin-Elmer). Relative luminescence readings were converted to % viability using untreated cells as controls. Data were fitted through nonlinear regression analysis, using log(inhibitor) versus response, variable slope, and 4-parameter fit equation using GraphPad Prism. Data were expressed as % relative cell viability versus the dose of free linker-warhead or ADC in nM, with error bars indicating the standard deviation (SD) of the quadruplicates.

### 2.9. ADC Binding to FcRn by Bio-Layer Interferometry 

FcRn binding to ADCs was determined by Bio-Layer Interferometry (BLI) on a ForteBio Octet QK 384. Biotinylated human FcRn protein was captured on streptavadin sensors. Antibody binding was measured at 1.5625, 3.125, 6.25, 12.5, 25, 50, and 100 nM. Binding was measured at both pH 5.8 and pH 7.4 to simulate the early endosomal and extracellular environments, respectively. Curve fits were performed with a one-to-one model using the ForteBio Data Analysis software.

### 2.10. LC-MS Analysis of ADCs

ESI-QTOF-MS was used to determine the intact molecular weight of the antibody and any variant isoform present. Chromatography separation was conducted through a Waters Acquity Ultra Performance liquid chromatography (UPLC) with diode array detection. The analytes were separated with an Agilent Zorbax 300 Diphenyl RRHD StableBond RP column (2.1 × 100 mm, 1.8 μm particle size and 30 nm pore size). The buffer A was 0.05% FA/0.05% TFA in water, and buffer B was 0.05% FA/0.05% TFA in acetonitrile. The separation was achieved by running a gradient of 30% B to 45% B over 15 min. The column temperature was maintained at 80 °C. The flow rate was 0.4 mL/minute. The outlet from the diode array detector of the UPLC was directed into a Waters Xevo ESI Q-TOF through a diversion control valve. The MS analysis was conducted in the positive ion mode with a capillary voltage of 3.3 kv, a cone voltage of 45 v, a source temperature of 150 °C, a desolvation temperature of 350 °C, and a desolvation gas flow rate of 800 L/hr. The data acquisition and analysis were achieved with the MassLynx v4.1 (SCN803). The combined raw ESI spectrum was background-subtracted prior to deconvolution through the MaxEnt 1 algorithm. For deconvolution consistency, the same m/z region was used for the same peak/species across all samples. For intact analysis, samples were diluted with HPLC-grade water to approximately 6 mg/mL. For reduced analysis, samples were diluted to approximately 2 mg/mL in 7.2 M guanidine hydrochloride, 100 mM tris, at pH 7.5. The diluted samples were heated at 50 °C for 15 min for denaturation, which was followed by reduction with 5 μL 0.5 M DTT at 50 °C for 15 min. The reduced samples were centrifuged at 12,000 RCF for 5 min. Approximately 80 μL supernatant was transferred to an HPLC sample vial for LC-MS analysis. 

## 3. Results and Discussion

### 3.1. Light-Chain Expression in E. coli

**Figure 2:**
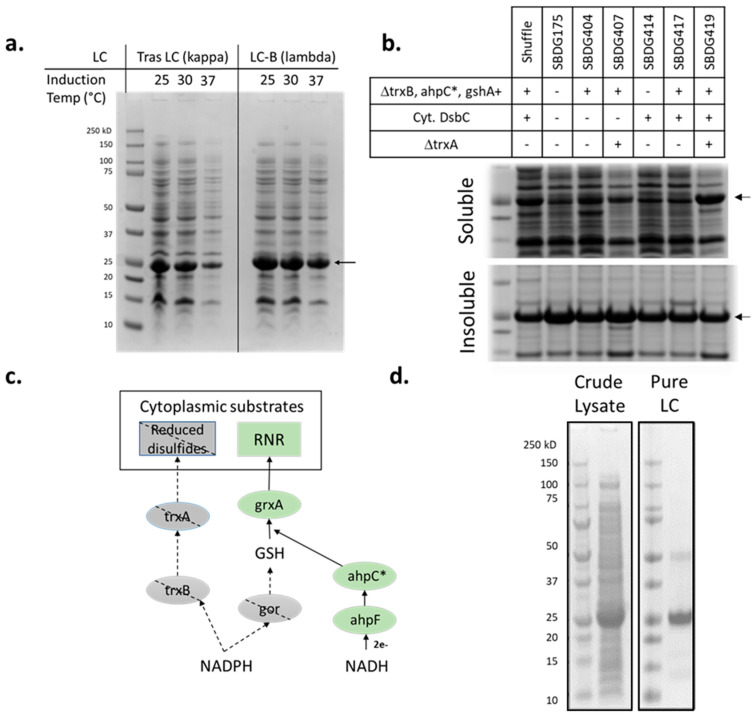
(**a**) Coomassie−stained SDS-PAGE gel showing soluble expression of two LC proteins in the Shuffle *E. coli* strain. Trastuzumab is a kappa LC while LC-B is a lambda LC. (**b**) Coomassie-stained SDS-PAGE gel showing soluble and insoluble expression of aCD74 LC in the Shuffle strain and strains described in this work, including the lineage preceding the final LC expression strain, SBDG419. A + indicates strains with the indicated genotype and a − denotes strains without. (**c**) Electron flow diagram in the thioredoxin/glutaredoxin systems responsible for maintaining reduced cytoplasmic cystines in *E. coli*. Dashed lines represent genetic knockouts responsible for establishing an oxidizing cytoplasm in the Shuffle strain and strain SBDG419. The mutant ahpC* enzyme can reduce glutathione (GSH) and maintains sufficient reducing capacity to preserve the activity of the essential enzyme ribonucleotide reductase (RNR). The trxA knockout was identified in the present work and found to be essential for soluble LC expression in the genetic background of Sutro’s strains. (**d**) Crude lysate of SBDG419 after expression of Trastuzumab LC in high density fed-batch cell culture and purified LC after a single-step purification using Kappa Select affinity resin.

To produce the LC protein as a reagent for cell-free protein synthesis reactions, we first set out to express it recombinantly in an *E. coli* host using standard techniques. Four LC genes representing molecules in active pre-clinical and clinical development at Sutro Biopharma were cloned into the pJ411 vector for cytosolic expression in *E. coli* cells under the control of the T7 promoter. Of the four LCs tested, three—Trastuzumab LC, an aCD74 LC, and LC-A—were kappa LCs and one—LC-B—was a lambda LC. Since the LC protein contains five cysteine residues, including two intramolecular disulfide bonds, we first tried expression in the Shuffle strain from NEB [25]. This commercially available strain for expressing disulfide-bonded proteins contains knockouts in the thioredoxin and glutaredoxin pathways, establishing an oxidizing cytoplasmic environment, and incorporates cytoplasmic expression of the disulfide isomerase DsbC. Figure 2 and Figure S1 show the soluble expression of the four different LC constructs in the shuffle strain grown at three temperatures post arabinose induction. All LCs show reasonable soluble expression at 25 °C, with decreasing expression as temperature increases. Cytoplasmic LC expression in an *E. coli* host with an oxidizing cytoplasm was demonstrated to be feasible for both kappa and lambda LC isotypes.

We next moved LC production into an *E. coli* strain derived from Sutro’s base reagent strain SBDG175 to facilitate large scale manufacturing and regulatory compliance. Strain SBDG175 has the glutaredoxin pathway removed through the Δ*gor* knockout and is glutathione deficient due to the Δ*gshA* knockout. Unlike the Shuffle strain, SBDG175 has an intact thioredoxin pathway resulting in a reduced cytoplasmic environment (Table 1). Accordingly, shake flask expression of the aCD74 LC in the SBDG175 strain background shows poor soluble expression compared with the oxidizing cytoplasmic environment in the Shuffle strain (Figure 2b, lanes 1 and 2). To obtain a strain with a more oxidizing cytoplasm, we first removed the gene for *trxB* in order to inactivate the thioredoxin pathway in addition to the already inactivated glutaredoxin pathway [26]. The Δ*trxB*Δ*gor* double knockout genotype is lethal since it lacks the ability to reduce the active site of the essential gene ribonucleotide reductase [27]; however, a suppressor mutation in the peroxidase *ahpC* has been identified, which establishes a minimal level of reduction to restore ribonucleotide reductase activity [28,29]. The mutant ahpC* acts through glutathione and *grxA*; thus, it is necessary to re-introduce the *gshA* gene, which restores the glutathione biosynthesis pathway. The *gshA* gene had previously been removed from extract and reagent strains to stabilize the amino acid cysteine in cell-free reactions [30] and can thus be replaced in this strain since it is intended for recombinant protein expression in vivo. Strain SBDG404 has the Δ*trxB* Δgor ahpC* *gshA*+ genotype establishing an oxidizing cytoplasm. While this strain demonstrates better LC soluble expression than the SBDG175 parent strain, it does not perform as well as the Shuffle strain (Figure 2b, lanes 1–3).

Cytosolic expression of the *E. coli* disulfide isomerase DsbC was next added as it is known to improve disulfide formation in both the Shuffle strain [25] and in cell-free protein synthesis reactions [15,16]. Surprisingly, the addition of cytosolic DsbC (Strain SBDG417) strain has a deleterious effect on soluble aCD74 LC expression (Figure 2b, lanes 3 and 6). This observation may be due to the presence of cryptic reductive pathways in the A19 *E. coli* lineage [31], which cytosolic DsbC expression either participates in directly or upregulates. We then proceeded to knock out *trxA* in the hopes of abolishing any residual activity in the thioredoxin pathway known to be responsible for the reduction of cytoplasmic disulfides (Figure 2c). Only when the *trxA* knockout is layered with the cytosolic DsbC expression in the Δ*trxB*, Δ*gor*, ahpC*, *gshA*+ background (strain SBDG419, Table 1) does the soluble aCD74 LC expression become improved to similar levels, as were observed in the Shuffle strain (Figure 2b, lanes 1 and7).

**Table 1:** List of strains used in the present work. Promoter sequences have been described previously [32]. ahpC* denotes a mutant ahpC enzyme [28,29].

Strain	Genotype	Citation
Shuffle^®^ T7	F’ *lac*, *pro*, *lacI^q^*/Δ(ara-leu)7697 araD139 fhuA2 lacZ::T7 gene1 Δ(phoA)PvuII phoR ahpC* galE (or U) galK λatt::pNEB3-r1-*cDsbC* (Spec^R^, *lacI^q^*) *ΔtrxB rpsL150*(Str^R^) *Δgor Δ(malF)3*	[25]
SBHS016	A19 Δ*tonA* Δ*tnaA* Δ*speA* Δ*endA ΔLAM* Δ*sdaA* Δ*sdaB* Δ*gshA Δgor RF1(N296K/L297R/L298R) araB::T7 galK::Pc0-2XDsbC met^+^*	[6]
SBDG175	SBHS016 *ΔompT araB::T7 galK::Pc0-2XDsbC*	this work
SBDG404	SBSH016 araB::T7 galK::Pc0-2XDsbC tnaA::Pc0-ahpC* trxB::Pwt-gshA	this work
SBDG407	SBDG403 ΔtrxA	this work
SBDG414	SBHS016 lacZ::Pmtl-2xlDsbC	this work
SBDG417	SBDG404 lacZ::Pmtl-2xlDsbC	this work
SBDG419	SBDG407 lacZ::Pmtl-2xlDsbC	this work

Strain SBDG419 was next used to produce LC on a gram scale. LC was expressed using high-density fed-batch *E. coli* culture in a 10-L bioreactor in defined I17 media. Soluble LC was then purified with Kappa Select affinity resin (Cytiva Life Sciences, Marlborough, MA, USA), which binds specifically to the CL constant region of kappa LCs. Figure 2d shows Coomassie-stained SDS-PAGE gels of Trastuzumab LC both in crude lysate and after a single affinity purification step. Typical LC titers after expression and purification are 3–9 g/L and final purity is >90%. An oxidized dimer species can be observed in purified LC preps at 10–30%, seen as a ~50 kDa band in Figure 2d. The C-terminal cysteine residue which would otherwise form an intermolecular bond with the HC in the native structure of an IgG is assumed to be responsible for this phenomenon. This dimer is resolved in the XpressCF+^®^ system through a combination of the DsbC prolyl isomerase enzyme and the oxidizing environment in the CFPS reaction. Accordingly, it has been observed that the amount of dimer in a LC preparation has no impact on the titer or final product quality of the resulting IgGs produced (data not shown).

### 3.2. Expression of Trastuzumab IgG with Prefabricated Light Chain in XpressCF+^®^

**Figure 3:**
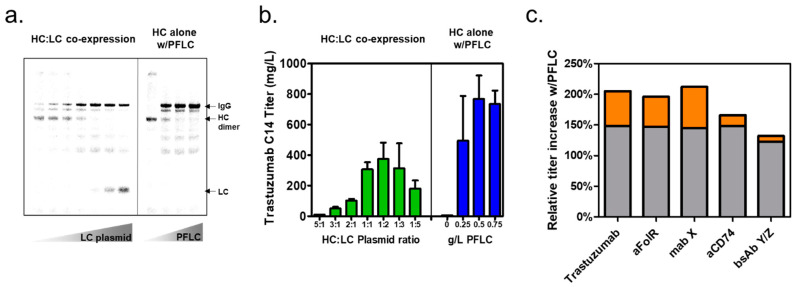
(**a**) C^14^ autoradiogram of SDS-PAGE gel showing Trastuzumab IgG expression in XpressCF+^®^ with either co-expression of the HC and LC or expression of the HC alone in the presence of the PFLC reagent protein. The HC/LC co-expression shows a plasmid ratio titration, which is required to identify the ratio of the two proteins which yields optimal IgG titer and assembly. The concentration of PFLC reagent was also titrated in the HC alone with PFLC expressions. (**b**) Trastuzumab IgG titer calculated from the autoradiogram in part (**a**). Green bars represent the HC/LC co-expression while blue bars represent the HC expression alone in the presence of the PFLC reagent. Error bars are calculated from SD of acid precipitable C14 detected by liquid scintillation counter (n = 2) (**c**) Comparison of the relative improvement of cell-free expression by C^14^ observed with the PFLC reagent compared to HC/LC co-expression for four mAbs and one bispecific antibody (FabFc:scFvFc format). The gray bars represent the theoretical titer improvement expected given a constant level of cell-free protein synthesis, folding, and assembly between the two reaction formats. Orange bars represent the additional titer increase observed, which is partially attributed the LC acting as a co-chaperone to improve HC solubility and final IgG folding and assembly.

Purified LC was next tested as a reagent to improve the expression of Trastuzumab IgG in the XpressCF+^®^ protein synthesis platform. Cell-free protein synthesis reactions were performed at a 100-µL scale and visualized with C^14^-Leucine labelling to compare IgG expression using the prefabricated LC (PFLC) reagent to expression, using HC/LC co-expression from two plasmids. Figure 3a,b shows a C^14^ autoradiogram of an SDS-PAGE gel and titer calculations from a cell-free reaction comparing the expression of Trastuzumab IgG with HC/LC co-expression to the expression of HC only, with the addition of the PFLC reagent. Expression of the Trastuzumab IgG using the PFLC enables a 105% increase in the productivity of the CF reaction relative to the reaction co-expressing HC and LC. 

The HC/LC plasmid ratio is a critical parameter for titer and assembly of IgGs expressed with two plasmids in a CFPS reaction, as demonstrated in Figure 3a. In the left lanes of the autoradiogram of the HC/LC co-expression gel, too little LC plasmid results in poor IgG assembly, and a lot of insoluble HC is detectible by acid precipitation of the total protein fraction. In the right lanes of the HC/LC co-expression gel, an abundance of LC plasmid results in excess production of LC, using resources that could otherwise be used to produce HC in the cell-free reaction, and reducing the final titer of correctly assembled IgG. The HC/LC plasmid ratio is a critical parameter for IgG expression using a two-plasmid co-expression system and is not only construct dependent but may also change when other inputs in the cell-free reaction are changed, for instance, reaction temperature, pH, extract %, extract lot, or plasmid lot. 

CFPS IgG expression using the PFLC reagent obviates the need for plasmid ratio titrations, creating a simplified and more robust process. The gel in Figure 3a shows the titration of PFLC concentration in a CFPS reaction expressing only the HC protein. The band for the LC is no longer observed in the C^14^ autoradiogram of the SDS-PAGE gel since C^14^-Leucine is only incorporated into proteins being synthesized in the CFPS reaction. Figure 3b demonstrates a 105% increase in titer over the HC/LC co-expression system when the PFLC is added to the CFPS reaction at or above 0.5 g/L. Optimal titer and assembly are achieved so long as the PFLC is supplied in sufficient excess. This approach leads to much more robust CFPS reactions since the optimal PFLC concentration is no longer construct or reaction condition dependent, eliminating the need for repetitive plasmid titrations as in the HC/LC co-expression reaction format. The elimination of plasmid ratio titrations is of particular benefit when screening large numbers of IgGs—for instance, as part of a high-throughput screening campaign—and greatly reduces the possibility of suboptimal assembly due to small perturbations in CFPS reaction conditions.

### 3.3. Prefabricated Light-Chain Titer Improvements for Additional Antibodies

Cell-free titer improvement with PFLC reagent addition was compared to HC/LC co-expression across four IgGs and a bispecific antibody (bsAb, Figure 3c) using 100-µL CFPS reactions with detection by C^14^. Three of the IgGs –Trastuzumab, an aFolR antibody, and mAb-X—use a common Trastuzumab LC; however, the aCD74 IgG and bsAb-Y/Z each use different LCs. Figure 3c shows the relative titer increase in CFPS using PFLC compared to the optimal titer achieved with HC/LC co-expression and demonstrates that all five products tested have a titer improvement in the PFLC format: 65–105% for the four IgGs tested and 32% for bsAb-X/Y. The simplest explanation for titer improvements with PFLC is that resources previously devoted to LC production can now be entirely utilized for producing the HC protein. HC accounts for two thirds of the mass of the final IgG product, so in the case of a constant rate of protein synthesis in the cell-free reaction, this simplified model predicts a 50% increase in IgG titer when switching from a HC/LC co-expression format to a PFLC format where 100% of CFPS protein synthesis resources are being dedicated to HC production. The titer improvement for the four IgGs tested were all greater than the 50% improvement predicted by this simplified model, suggesting that additional factors contributed to the titer increases observed. The titer increases predicted from the constant protein synthesis model are represented by the gray bars in Figure 3c while the excess titer improvements observed experimentally are represented by orange bars. Though the reallocation of CFPS translation resources to producing only the HC protein appears to be responsible for the bulk of the IgG titer increase, the larger than predicted titer increases observed with PFLC are likely to have arisen from several effects. First, we find that the optimal plasmid ratio for cell-free produced IgGs using HC/LC co-expression often requires excess LC expression. The addition of the PFLC as a reagent eliminates the need for this imbalance in subunit expression, allowing more overall protein synthesis resources to be dedicated to HC, resulting in higher final IgG titers. Second, we expect that the addition of excess PFLC reagent to the CFPS reaction results in a higher degree of soluble HC expression due to the ability of the LC to act as a co-chaperone, which aids in HC folding, improving overall IgG assembly. The HC CH1 domain is known to be intrinsically disordered unless associated with either the Hsp70 ER chaperone BiP or with its cognate LC [33]. This hypothesis is consistent with the observation that HC expressed alone in the absence of LC has limited solubility, as well as the previous result demonstrating that IgG titers in cell-free proteins can be improved by LC pre-synthesis, adding the LC plasmid prior to the HC plasmid in order to facilitate correct folding and assembly [14].

We have also demonstrated the benefits of PFLC for an scFvFc/FabFc bispecific antibody (bsAb) containing only a single LC protein. This molecule, bsAb-Y/Z, contains three protein subunits, an svFv-Fc, a HC, and single LC, with correct chain pairing enforced by steering mutations in the Fc region [34]. Figure 3c shows a 32% titer improvement for this bsAb compared with the 25% improvement predicted from the simplified constant protein expression model. This suggests that reallocation of CFPS resources is responsible for most of the titer improvement observed for the PFLC-expressed bsAb. In the case of a bsAb, the addition of PFLC to the CFPS reaction reduces the number of plasmids required from three to two. This antibody format is particularly sensitive to small changes in plasmid ratio, so the reduction in the number of degrees of freedom in plasmid ratio titration represents a significant improvement in robustness and a simplification of the overall CFPS process. 

### 3.4. Demonstration of Prefabricated Light-Chain Titer Improvements at Larger Reaction Formats

**Figure 4:**
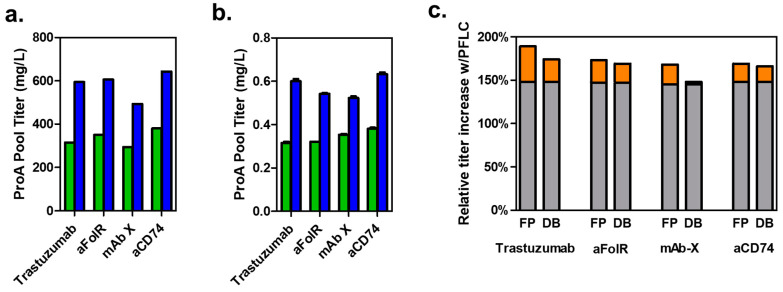
(**a**) CFPS expression of four IgGs in a 1-mL flowerplate reaction format with either HC/LC co-expression (green bars) or the expression of HC alone with PFLC reagent addition at 0.5 mg/L (blue bars). These reactions were performed in a plate-based format with temperature control but without pH or DO control. (**b**) CFPS expression of four IgGs in a 250-mL stirred tank reaction format with either HC/LC co-expression (green bars) or the expression of HC alone with PFLC reagent addition at 0.5 mg/L (blue bars). These reactions had temperature, agitation, DO, and two-sided pH control. Data represent mean ± SD for n = 2 titer assays from a single reaction. (**c**) Comparison of the relative improvement of cell-free expression by flower plate (FP) or Dasbox (DB) observed with the PFLC reagent compared to HC/LC co-expression for the four IgGs tested. The gray bars represent the theoretical titer improvement expected given a constant level of cell-free protein synthesis, folding, and assembly between the two reaction formats. Orange bars represent the additional titer increase observed, which is partially attributed to the LC acting as a co-chaperone to improve HC solubility and final IgG folding and assembly.

We next demonstrated that the titer improvement seen at the 100-µL scale was observed at larger scales and in different reaction formats. Figure 4a,b shows the results of CFPS with HC/LC co-expression and the expression of HC alone with the PFLC reagent in both a 1-mL plate and 250-mL stirred tank reaction format. The titer for both reaction formats was determined by high-throughput purification on 20 µL Protein A Phytip with titer determination by A_280_ absorbance. The 1-mL (Figure 4a) reactions were performed in a flowerplate (m2p-labs) format and had no pH or DO control. The 250-mL stirred tank reactions (Figure 4b) were performed in a DASbox (Eppendorf) with DO and two-sided pH control. All four IgGs show a titer improvement when HC is expressed with the PFLC reagent in both reaction formats, with relative titer improvements of 48–98% compared with the HC/LC co-expression experiments (Figure 4c). All molecules tested show a titer improvement of at least 50%, consistent with the hypothesis that the primary mechanism is the relocation of CFPS translation resources to HC production (gray bars, Figure 4c). In most cases, additional titer improvement above 50% is also observed (orange bars, Figure 4c).

We have previously shown that cell-free titers scale well from 100-µL research scale to 1000-L manufacturing scale reactions [24] and the titer improvements from the PFLC process presented here are predicted to scale similarly. The stirred tank bioreactor demonstration of the titer benefits from the PFLC process are particularly relevant since this reaction format serves as a direct scale-down model of the large-scale 1000-L manufacturing process. These titer increases should result in an overall net decrease in the cost of goods for ADC manufacturing in the XpressCF+^®^ CFPS platform. The cost of development and manufacturing of the new PFLC reagent for the cell-free reaction are predicted to be offset by titer improvements in the final product, as well as reductions in the cost of plasmid and efficiencies in research and development with the improved PFLC process.

### 3.5. Comparability of ADCs Produced with Prefabricated Light Chain

Site-specific ADCs were next produced using either PFLC or HC/LC co-expression in order to demonstrate comparability of biophysical properties and biological activity. For these experiments, we worked with the nnAA *p*-azidomethyl phenylalanine (pAMF), which was previously enabled for ADC production with CFPS [5], although the PFLC strategy should be compatible with other nnAA chemistries. Two IgGs, aCD74 and mAb-X, were scaled up in 0.25-L stirred tank bioreactors, captured with Protein A affinity chromatography, and polished to homogeneity by preparative SEC. Strain-promoted azide-alkyne click chemistry (SPAAC) reactions were then used to conjugate a DBCO-maytansine drug linker, SC236 [35], to the azidio group on the pAMF nnAA creating the DAR2 and DAR4 site-specific ADCs aCD74 and mAb-X, respectively. Conjugates showed no differences in purity by analytical SEC, and all ADCs were conjugated at >95% efficiency by MALDI-TOF MS (Table 2). Intact mass LC-MS analysis demonstrates that ADCs made by HC/LC co-expression or PFLC have mass differences within 5 Da (Figures S2 and S4), while reduced LC-MS analysis shows that the LC components of each ADC have mass differences within 1 Da (Figures S3 and S5). ADCs produced in CFPS using either PFLC or HC/LC co-expression are comparable by standard product quality assessments investigating assembly, purity, DAR, and ID by LCMS.

**Figure 5:**
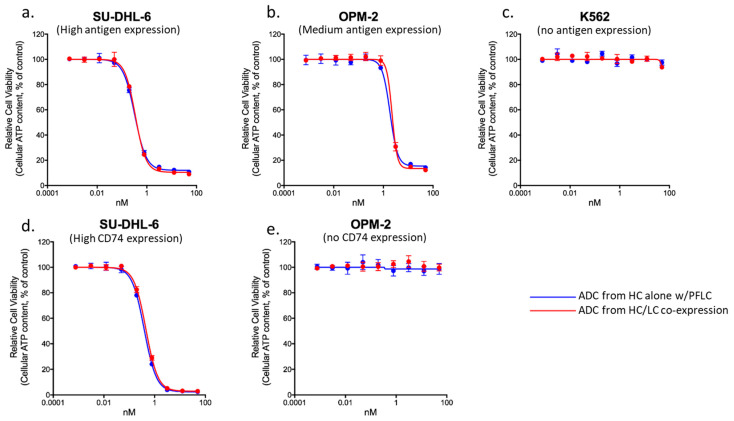
Cell killing results for ADC-X (**a**–**c**) and aCD74 (**d**,**e**) ADCs. ADC-X was conjugated to the DBCO-PEG-maytansine drug-linker at a DAR of four and was tested on cell lines with high (**a**) medium (**b**) and no (**c**) antigen expression. aCD74 SC236 drug-linker at a DAR of two was tested on cell lines with high (**d**) and no (**e**) CD74 expression. Data represent mean ± SD (n = 4).

PFLC and HC/LC co-expression-produced ADCs were further evaluated in an in vitro cell killing assay to demonstrate their functional equivalence. ADC-X, the conjugate of mAb-X, was tested in cell lines with high, medium, and no antigen expression (Figure 5a–c). The aCD74 ADC was tested in high and no antigen expressing cell lines (Figure 5d,e). The cell killing curves for both PFLC and HC/LC co-expression-produced ADCs are superimposable for all cell lines and antigens tested, indicating that cell killing activity is unaffected by the CFPS production system used.

Additional characterization of ADC binding to the neonatal Fc receptor, FcRn, was carried out by Bio-Layer Interferometry (BLI). ADC binding to the FcRn receport protects from degradation and has been demonstrated to be an important determinant of efficacy, safety, and pharmacokinetics (PK) [36]. aFolR ADCs were prepared by conjugating the SC239 DBCO-hemiasterlin drug linker to IgG produced in CFPS using either two-plasmid co-expression or PFLC [37]. Comparison of the BLI data (Figure S6) and the resulting kinetic constants from fitting with a one-to-one model (Table 3) demonstrate that binding to FcRn at pH 5.8, the physiologically relevant pH, is identical for proteins produced by CFPS, irrespective of the method of LC production. As expected, neither production method showed FcRn binding at pH 7.4. This suggests that the PFLC will not have a significant impact on the in vivo half-life or PK profile of ADCs produced in CFPS. The comparability data presented suggest that the PFLC process will not have a significant impact on CFPS produced ADCs in terms of product quality, efficacy, or pharmacokinetic properties.

## 4. Conclusions

In the present work, we present a method for improving antibody titer and manufacturability in the XpressCF+^®^ system. We have engineered a cell line with an oxidizing cytoplasm capable of LC expression at the 3–9-g/L scale. Prefabricated LC protein was then added as a protein reagent to CFPS reactions expressing the antibody HC, and a 50–100% increase in titer was observed relative to CFPS reactions co-expressing both the HC and LC antibody subunits. The primary mechanism for this titer increase is believed to be through the reallocation of the CFPS translation machinery to focus on HC production. This method was validated for five antibodies and demonstrated at scales from a 100-µL microplate to a 0.25-L stirred tank bioreactor. Antibodies produced in CFPS using PFLC were indistinguishable from those produced using HC/LC co-expression by both biophysical and functional assays. This approach combines the strength of in vivo *E. coli* expression (inexpensive manufacturing of recombinant proteins with high titer) with cell-free protein synthesis (an environment capable of incorporating multiple nnAAs in properly folded and assembled IgGs). The benefits of this system are numerous; the PFLC reagent increases titers, streamlines the CFPS workflow, and makes the CFPS reactions more robust when compared to the conventional HC/LC co-expression approach. Development of the hybrid PFLC cell-free platform described in this work improves the competitiveness of cell-free manufacturing for site-specific ADCs, allowing patients expanded access to these promising new molecules.

## Figures and Tables

**Table 2:** Comparison of product quality between ADCs of aCD74 and ADC-X produced in CFPS reactions with a HC/LC co-expression format or a HC expression with PFLC reagent format. Analytical SEC results report the relative amounts of High Molecular Weight (%HMW), Monomer, and Low Molecular Weight species (%LMW) by integration of the HPLC chromatogram at 280 nm. Conjugation efficiencies by MALDI-TOF MS are reported as Drug Antibody Ratio (DAR) and conjugation efficiency (% conj).

		aCD74 ADC		ADC-X	
Analytical SEC	% HMW	1%	1%	1%	2%
	% Monomer	95%	98%	96%	95%
	% LMW	4%	1%	3%	3%
MALDI-TOF MS	DAR	1.9	1.9	3.9	3.9
	% conj	95%	95%	98%	98%

**Table 3:** Comparison of kinetic parameters from SPR binding of aFolR ADC to FcRn.

Sample	Rmax	k_on_ (M^−1^s^−1^)	k_off_ (M^−1^)	*K_D_ (M)*
*aFolR (HC/LC)*	2.4	4.3 × 10^5^	1.2 × 10^−3^	2.8 × 10^−9^
*aFolR (PFLC)*	2.4	3.7 × 10^5^	1.2 × 10^−3^	3.2 × 10^−9^

## Data Availability

The data presented in this study are available on request from the corresponding author.

## Supplementary Materials

The following supporting information can be downloaded at: https://www.mdpi.com/article/10.3390/bioengineering10030304/s1, Figure S1: SDS-PAGE gel of shake-flask LC expression, Figures S2–S5: Reducing and non-reducing deconvoluted LCMS spectra of ADC-X and aCD74 ADC. Figure S6: BLI response curves of aFolR ADC binding to FcRn.
