# Supplementary material for: An Integrated In Vivo/In Vitro Protein Production Platform for Site-Specific Antibody Drug Conjugates
Source: Bioengineering (Basel). 2023 Feb 28;10(3):304. doi: 10.3390/bioengineering10030304 (PMC10045668; doi:10.3390/bioengineering10030304)
Supplement: Supplementary file 1 [file bioengineering-10-00304-s001.zip › bioengineering-2225798-supplementary.pdf]

An Integrated In Vivo/In Vitro Protein Production Platform for Site-Specific Antibody Drug Conjugates

Jeffrey Hanson[1], Dan Groff[1], Abi Carlos [1], Hans Usman[1], Kevin Fong[1], Abbigail Yu[1], Stephanie Armstrong[1], Allison Dwyer[1], Mary Rose Masikat[1], Dawei Yuan[1], Cuong Tran[1], Tyler Heibeck[1], James Zawada[1], Rishard Chen[1*], Trevor Hallam[1], and Gang Yin[1]

[1]Sutro Biopharma Inc, 111 Oyster Point, South San Francisco, CA 94080

[*]Current Address: Good Meat, 300 Wind River Way, Alameda, CA94501

Supplemental Information

Supplemental Figures

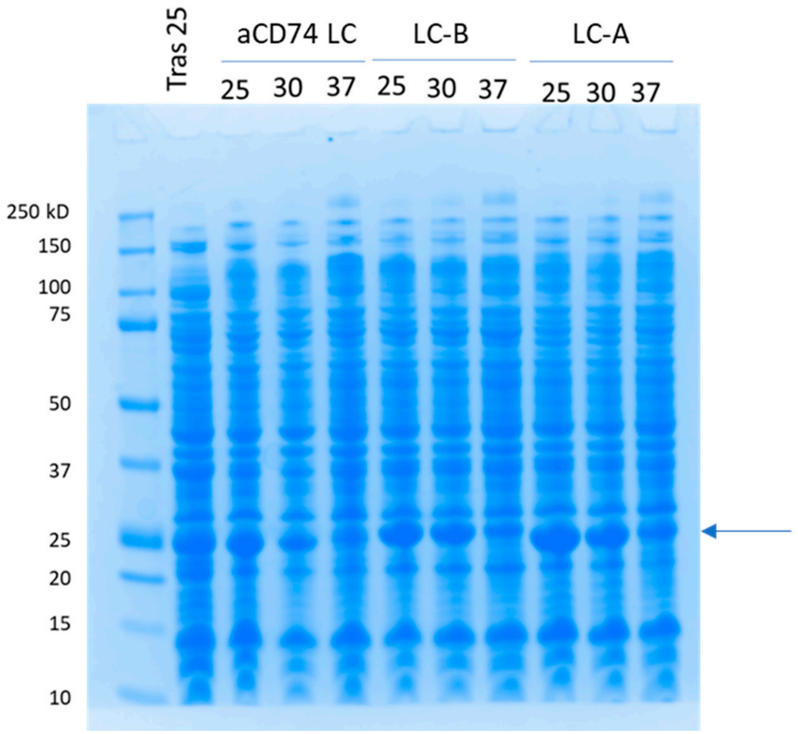

Figure S1 Coomassie stained SDS-PAGE Gel showing soluble LC expression in the shuffle strain in crude lysate for three LCs at three induction temperatures each. Expression was done in shake flask with overnight induction. LC band is the most prominent protein in the lysate, indicated with an arrow. Best soluble expression was observed at 25C.

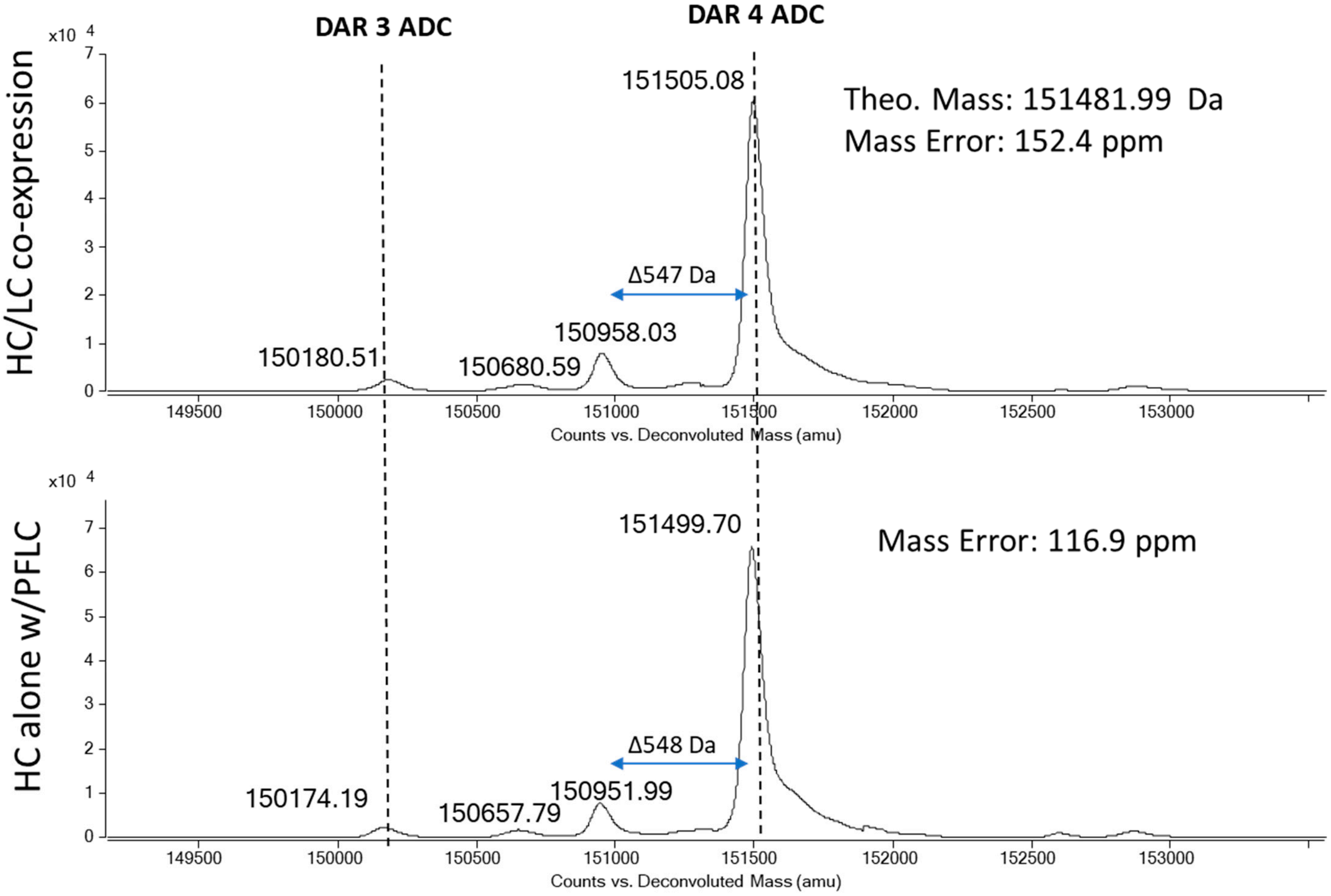

Figure S2 Deconvoluted intact mass LC-MS spectra of ADC-X made with HC/LC co-expression and PFLC.

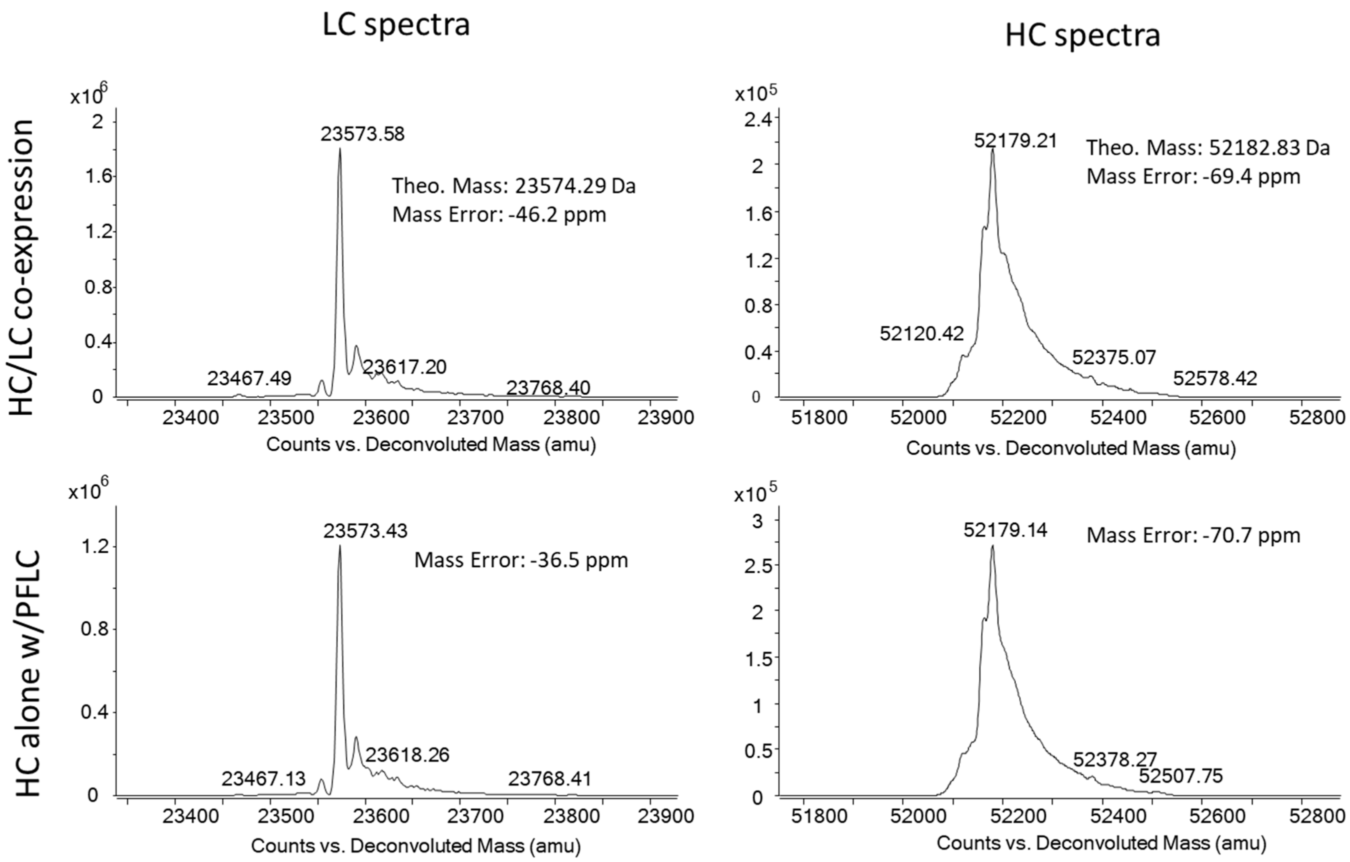

Figure S3. Deconvoluted reduced LC-MS spectra of ADC-X made with HC/LC co-expression and PFLC.

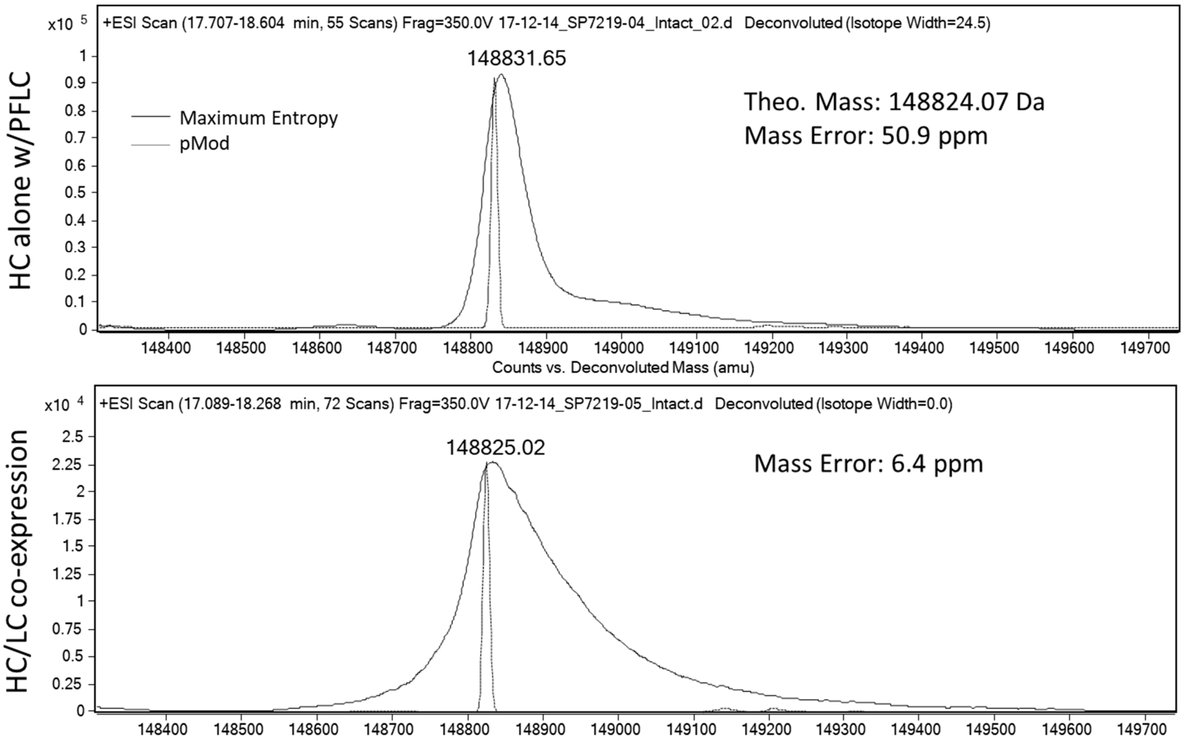

Figure S4. Deconvoluted intact mass LC-MS spectra of aCD74 ADCs made with HC/LC co-expression and PFLC.

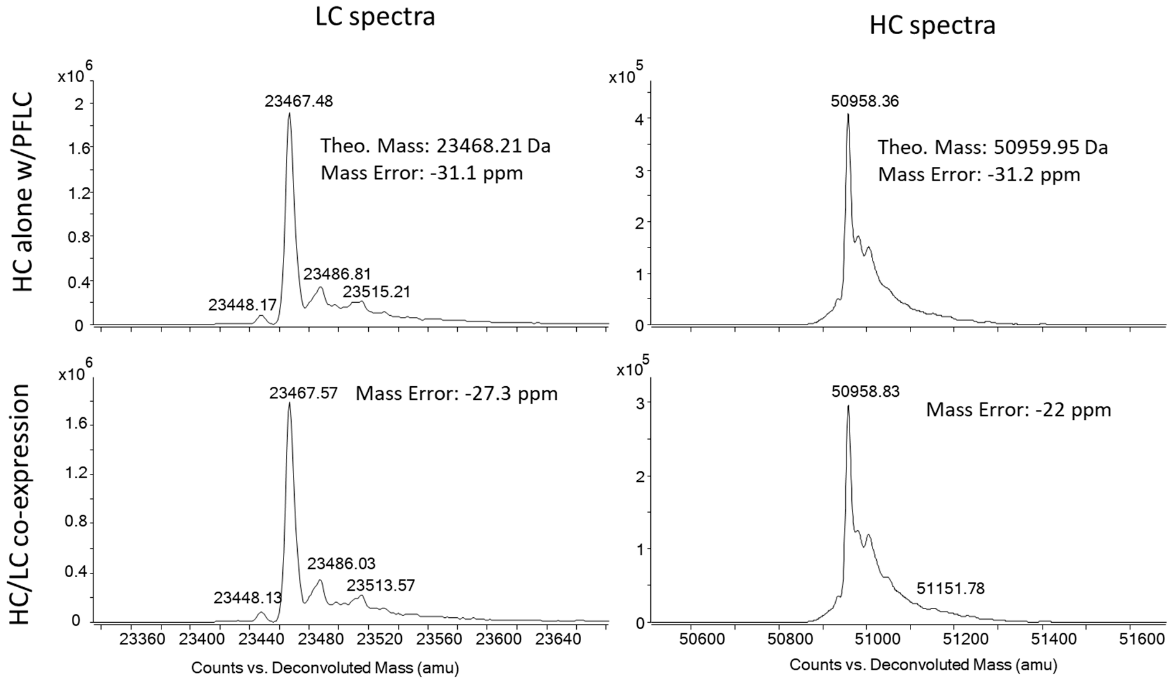

Figure S5. Deconvoluted reduced LC-MS spectra of aCD74 ADCs made with HC/LC co-expression and PFLC.

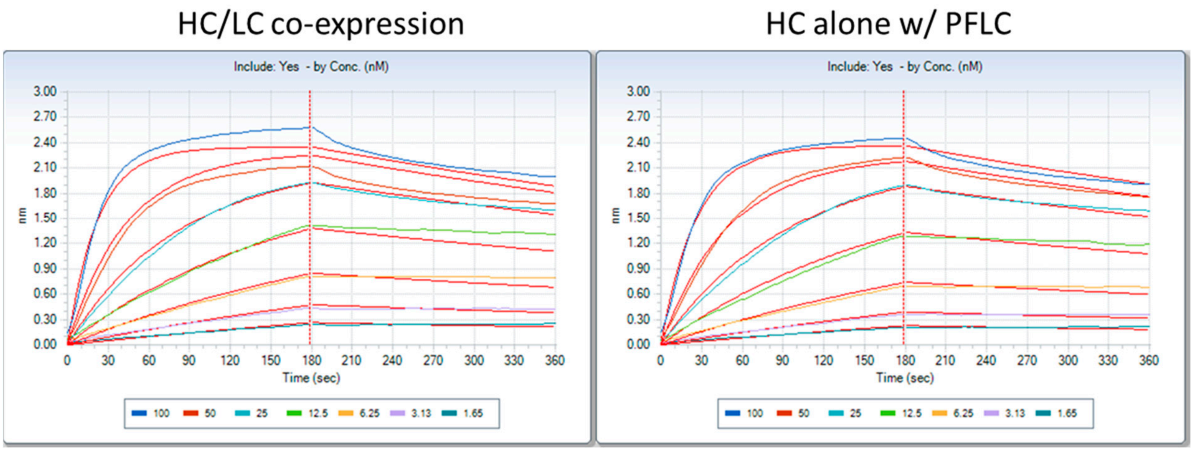

figure S6. aFolR ADC binding to FcRn by Bio-Layer Interferometry (BLI) comparing ADCs made with HC/LC co-expression and PFLC **.**
